# Graphene Oxide–Silver Nanoparticles Nanocomposite Stimulates Differentiation in Human Neuroblastoma Cancer Cells (SH-SY5Y)

**DOI:** 10.3390/ijms18122549

**Published:** 2017-11-28

**Authors:** Sangiliyandi Gurunathan, Jin-Hoi Kim

**Affiliations:** Department of Stem Cell and Regenerative Biotechnology, Konkuk University, Seoul 05029, Korea

**Keywords:** neuroblastoma, luciferin, differentiation, apoptosis, neuronal markers, stem cell markers

## Abstract

Recently, graphene and graphene related nanocomposite receive much attention due to high surface-to-volume ratio, and unique physiochemical and biological properties. The combination of metallic nanoparticles with graphene-based materials offers a promising method to fabricate novel graphene–silver hybrid nanomaterials with unique functions in biomedical nanotechnology, and nanomedicine. Therefore, this study was designed to prepare graphene oxide (GO) silver nanoparticles (AgNPs) nanocomposite (GO-AgNPs) containing two different nanomaterials in single platform with distinctive properties using luciferin as reducing agents. In addition, we investigated the effect of GO-AgNPs on differentiation in SH-SY5Y cells. The synthesized GO-AgNPs were characterized by ultraviolet-visible absorption spectroscopy (UV-vis), X-ray diffraction (XRD), scanning electron microscopy (SEM), transmission electron microscopy (TEM) and Raman spectroscopy. The differentiation was confirmed by series of cellular and biochemical assays. The AgNPs were distributed uniformly on the surface of graphene oxide with an average size of 25 nm. As prepared GO-AgNPOs induces differentiation by increasing the expression of neuronal differentiation markers and decreasing the expression of stem cell markers. The results indicated that the redox biology involved the expression of various signaling molecules, which play an important role in differentiation. This study suggests that GO-AgNP nanocomposite could stimulate differentiation of SH-SY5Y cells. Furthermore, understanding the mechanisms of differentiation of neuroblastoma cells could provide new strategies for cancer and stem cell therapies. Therefore, these studies suggest that GO-AgNPs could target specific chemotherapy-resistant cells within a tumor.

## 1. Introduction

Neuroblastoma is the most common solid tumor of infancy and it accounts for more than 7% of childhood cancers and 15% of cancer-related childhood deaths [1]. There are about 700 new cases of neuroblastoma each year in the United States. Neuroblastoma is an embryonic malignancy of early childhood originating from neural crest cells and it is most common extracranial solid tumor that arises from the developing sympathetic nervous system occurring in childhood with different clinical presentation [2,3]. The poor diagnosis of neuroblastoma presents at the age of ≥18 months and leads to disseminated disease as metastatic processes in liver, bone marrow, skin and several other organs [4]. Although diagnosed very well, about half of all diagnosed cases are classified as high-risk (HR) for disease relapse, while overall survival rates still show only modest improvement, less than 40% at five years [1]. Therefore, over the past decade, understanding and treatment of this disease has advanced tremendously. The HR patients are being treated by various therapeutic approaches including chemotherapy, surgery, and radiotherapy, immunotherapy with anti-disialoganglioside (GD2) antibody and differentiation therapy with 13-*cis* retinoic acid [5]. Neuroblastoma arises from the neural crest cell precursors of the sympathetic nervous system, which fail to differentiate and are the best platform for differentiation therapy [6,7].

Most high-risk neuroblastomas patients, around 50–60%, initially respond to chemotherapy but ultimately relapse, acquiring drug resistance. In addition, conventional therapy such as chemo- and radiotherapy has undesired side effects such as killing non-cancerous cells. Differentiation agents seem to be alternative treatments that tend to have less toxicity than conventional cancer treatments [4]. Therefore, differentiation therapy holds great promise for cancer treatment. Differentiation therapy seems to be an attractive approach for the treatment of advanced or aggressive malignancies in which the malignant cells begin the process of maturation and differentiation into mature cells. Previous studies reported that tumor regression is induced by several factors including nutrient conditions, chemicals, and genetic processes by the process of cancer cells into normal cells by the process of differentiation [8,9]. The differentiation stage of tumors is a critical and prognostic parameter in histopathological analysis of solid malignancies and is strongly associated with tumor behavior, and generally an immature tumor is more aggressive than the more differentiated counterpart. A high degree of differentiation serves a better prognosis than a low degree in prognostic implications in cancer, which help to understand the cellular and molecular mechanisms of cancer [10]. The better approach for cancer treatment is targeting proliferating cells because these progeny cells will have enough divisions to kill a patient and differentiation therapy could force cancer stem cells to differentiate and lose their self-renewal property [11]. Differentiation is an important phenomenon in cancer cells, and differentiation therapy holds great promise for cancer treatment [12]. Chemical compounds and androgen deprivation induce differentiation of neuroendocrine cells in prostate cancer [8,9]. The significance of differentiation of cancer cells into normal tissue cells, which contributes to tumor regression, is induced by some factors, including genetic processes, nutrient conditions, and chemicals [13]. Interestingly, silver nanoparticles (AgNPs) induce neuronal differentiation via modulation of reactive oxygen species, phosphatases, and kinase signaling pathways in SH-SY5Y cells [14]. Furthermore, substrates coated with AgNPs, serving as favorable anchoring sites, significantly enhance neurite outgrowth [15]. These studies suggest that restoration of normal function or differentiated phenotypes in cancer cells are related to tumor suppressive function.

Graphene oxide has immense interest in several biomedical applications as biosensors, drug carriers, antibacterial, antiplatelet, and anticancer agents and scaffolding material for tissue engineering due to its potential properties such as large surface areas, abundant functional groups, and high water solubility [16]. A study suggested that GO significantly enhanced the differentiation of SH-SY5Y induced-retinoic acid (RA) by enhancing expression micro-tubule associated protein 2 (MAP2) [17]. Graphene and graphene related nanomaterials exhibited biocompatibility/toxicity with various cell lines including primary mouse embryonic fibroblast cells, human breast cancer cells, and human embryonic kidney (HEK) 293 cells [18,19,20]. Furthermore, graphene and graphene family materials are known to support cellular attachment as scaffolding agent, as well as to induce proliferation and differentiation [21,22]. Reduced graphene oxide (rGO) films are biocompatible and induce neurite genesis of PC12 cells, and graphene substrates promote the neurite sprouting and outgrowth of mouse hippocampal cells [23,24]. Furthermore, GO suspension induces osteogenic differentiation of human mesenchymal stem cells (MSCs) and enhances the differentiation of mouse embryonic stem (ES) cells to both primitive and definitive hematopoietic cells [25,26].

Selections of appropriate in vitro model systems are essential to provide translational models for human diseases. Maximum in vitro experimental studies were conducted in primary neuronal cultures from rats and mice; however all of these models show lack of sufficient human component, dissimilar characteristics that are not identical to human beings, unstable karyotyes and problems for studies of gene expression and reproducible studies of cell signaling [27]. Therefore, developing more consistent models of human neurological disease is necessary. SH-SY5Y neuroblast-like cells have a stable karyotype consisting of 47 chromosomes, and can be differentiated from a neuroblast-like state into mature human neurons through a variety of different mechanisms induced by diversity of differentiating agents including RA, phorbol esters, and specific neurotrophins such as brain-derived neurotrophic factor (BDNF) [27]. SH-SY5Y cells can be induced to differentiate into neuron-like phenotypes in vitro by several agents such as retinoic acid, vasoactive intestinal peptide, graphene oxide and silver nanoparticles [14,28,29,30,31,32]. The neuronal differentiation process includes morphological, biochemical, and electrophysiological changes as the cell progresses to a mature neuron phenotype [31]. However, no studies report, to the best our knowledge, the synergistic effect of graphene oxide and silver nanoparticles in the aspects of neuronal differentiation in SH-SY5Y cells. The interesting aspects of graphene oxide–silver nanoparticle nanocomposites are having two different physical and chemical properties in single platform. Considering the literature, we designed the following objectives: (1) synthesis and characterization of graphene oxide–silver nanoparticle nanocomposites using luciferin as reducing and stabilizing agent; (2) evaluation of the potential effect of biomolecule assisted graphene oxide–silver nanoparticle nanocomposites on differentiation in human neuroblastoma cancer cells (SH-SY5Y) using batteries of cellular and biochemical assays; and (3) investigation of the mechanism of induction of differentiation uisng graphene oxide–silver nanoparticle nanocomposites by analysis of expression of genes involved in differentiation.

## 2. Results and Discussion

### 2.1. Synthesis and Characterization of GO and GO-AgNPs Using Luciferin

**Figure 1:**
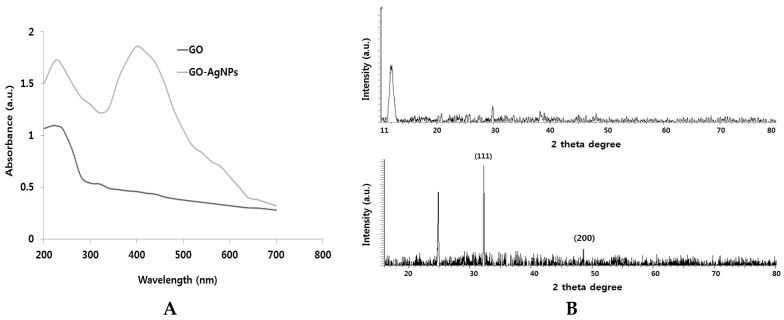

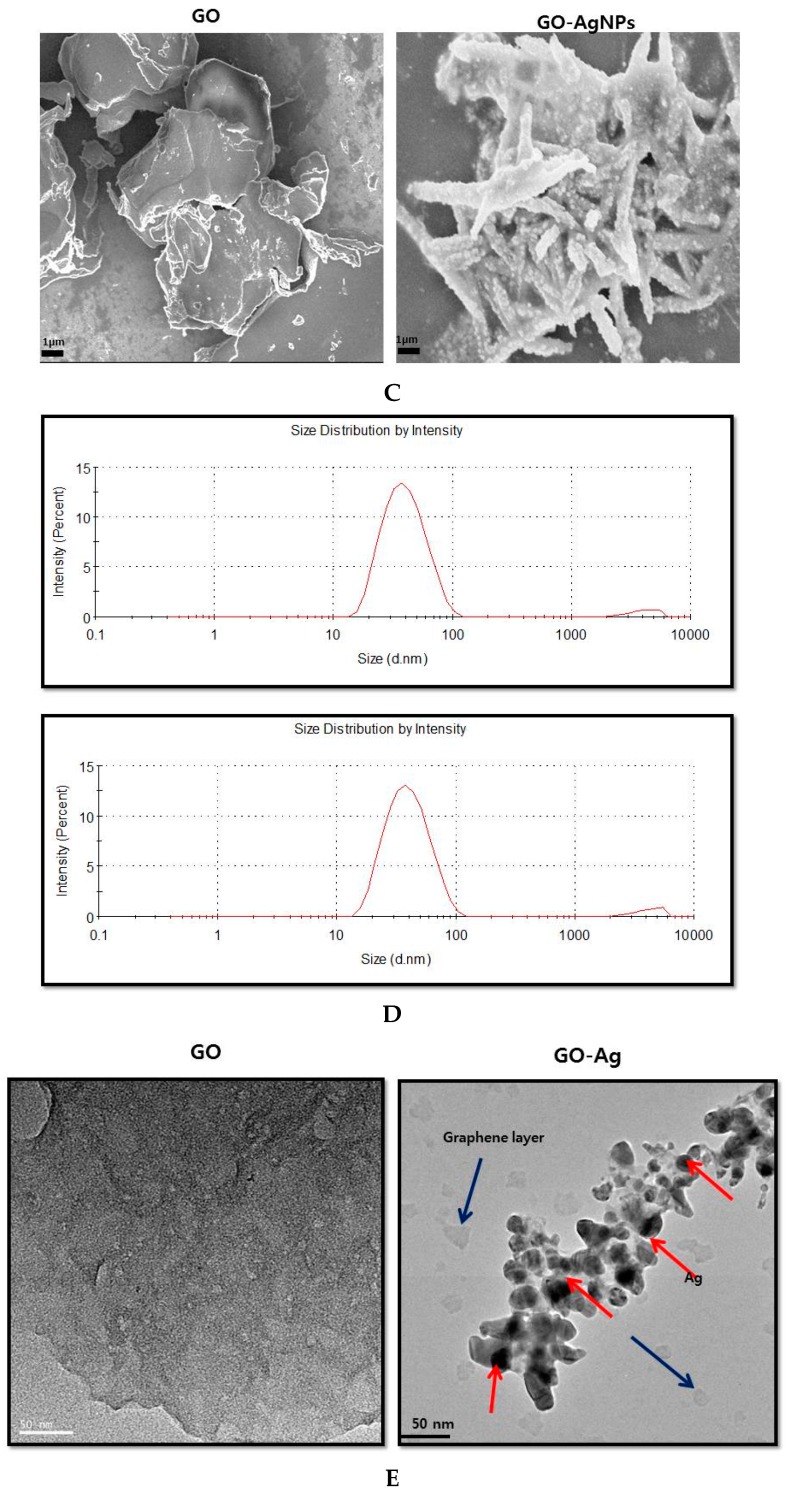

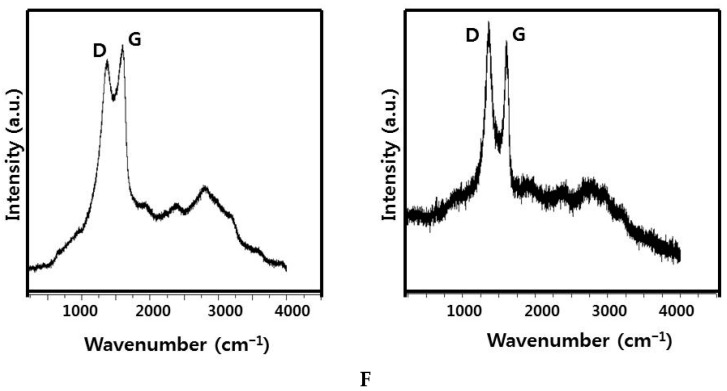
Synthesis and characterization of graphene oxide and graphene oxide–silver nanoparticle nanocomposite using lycopene. (**A**) Ultraviolet-visible spectroscopy of graphene oxide (GO) exhibit a maximum absorption peak at ~230 nm corresponding to the π–π transitions of aromatic C-C bonds. The absorption peak for GO-AgNPs exhibit peak at 230 nm and new peak at 410 nm is observed after deposition of AgNPs on the GO surface; the band at 410 nm in the absorption spectrum of the GO-AgNPs nanocomposite is attributed to surface plasmons and the presence of AgNPs; (**B**) X-ray diffraction (XRD) images of GO and GO-AgNPs; (**C**) scanning electron microscopy (SEM) images of GO and GO-AgNPs (**D**) Dynamic light-scattering (DLS) spectra of GO and GO-AgNPs dispersions. At least 200 particles were measured for each sample to obtain the size distribution; (**E**) transmission electron microscopy (TEM) images of GO and GO-AgNPs; (**F**) Raman spectroscopy images of GO and GO-AgNPs. At least three independent experiments were performed for each sample and reproducible results were obtained. The data present the results of a representative experiment.

Graphene is a single atomic plane layer of graphite, and graphene oxide is prepared from exfoliation of graphite oxide. It can produce different dispersion forms with different long-term stabilities and single layer GO thickness [33]. The UV-vis spectra of aqueous GO dispersions are presented in Figure 1A. GO shows typical characteristics features such as a shoulder at ~300 nm, corresponding to an n–π* plasmon peak and a feature at 230 nm corresponding to a *π*–π* plasmon peak. The intensity and position of this feature vary between samples depending on type of solvent used, sonication time and several other factors [33]. GO-AgNPs were also confirmed by UV-visible spectra. As shown in Figure 1A, the AgNPs attached on the layer of graphene show a typical characteristic peak at 410 nm, which was consistent with the surface plasmon resonance phenomena of AgNPs formulation [34], while GO exhibited a typical peak around 230 nm corresponding to the C=C aromatic bonding.

The crystalline nature of GO and GO-AgNPs was further confirmed using XRD. As shown in Figure 1B, the graphene oxide powder exhibited a sharp diffraction peak (001) at 2θ = 11.7°, which was assigned to the (001) reflection of GO. These results indicated the successful synthesis and significantly corroborated with previous reports of GO synthesis [33]. GO-AgNPs composite shifted the peak values at about 32.8° and 48.3°, which are assigned to the (111) and (200), crystal lattice planes of face-centered cubic (fcc) of Ag nanoparticles, respectively, while the peak of GO disappeared, which was reported because the metal nanoparticles attached onto the inlayers may lead to the sheeting the signals of graphene oxide peaks.

The surface morphology of GO and GO-AgNPs was examined by SEM. As shown in Figure 1C, the GO sheets are uniformly covered with AgNPs of different sizes of the AgNPs appear as white spots on the surface of graphene sheet. The surface of GO samples exhibited several layers of aggregated and square-shaped crumpled sheets closely associated with each other to form a continuous conducting network [18,19,20]. The edges of the GO sheets become crumpled, folded, and closely restacked and the surface of GO exhibits a soft carpet-like morphology, possibly because of the presence of residual H_2_O molecules and hydroxyl or carboxyl groups [18,19,20]. In contrast, GO-AgNPs exhibit transparent rippled silk-like waves or a flaky, scale-like, appearance that looks like tentacles of coral-reef structure. Graphene nanosheets showed coarse and hairy surfaces with blurry flake edges [18,19,20]. Figure 1C shows typical SEM images of the as-prepared GO-AgNPs hybrids; well-dispersed AgNPs are deposited on the graphene. The AgNPs are well separated from each other and randomly distributed on the graphene sheets as spacers keeping the neighboring sheets separate.

Toxicity or beneficial effects of any nanoparticle depend on size, morphology, size distribution, particle composition, surface area, surface chemistry, and particle reactivity in solution. DLS was used to determine the size of the particle in solution, which is very useful technique to evaluate particle size and size distribution of nanomaterials in solution [19,20]. Therefore, we examined the size distribution of GO and GO-AgNPs. The size distribution of both GO and GO-AgNPs was between 10 and 100 nm, with an average size of 50 nm (Figure 1D). The size distribution of the AgNPs and GO-AgNPs nanoparticles suspended in water seems to be slightly larger in size than the size in TEM analysis because the hydrodynamic size is usually affected by surface charge of particles.

TEM is one of the most valuable tools to directly analyze structural information of nanoparticles such as size and morphology under high vacuum conditions with a dry sample. TEM micrographs of the GO-AgNPs revealed distinct, uniformly spherical shapes that were well separated from each other on the surface of graphene sheets. The average particle size was estimated from measuring more than 200 particles from TEM images and showed particle sizes between 5 and 30 nm with an average size of 25 nm (Figure 1E). As shown in Figure 1E, GO shows the typical flaky structure like the wrinkle, wavy, and transparent pure graphene oxide, whereas GO-AgNPs were filled with AgNPs particles on the surface of graphene layer. AgNPs were displayed obviously as dots on the layer of graphene layers, while the dots tend to aggregate with each other to form high ratio volume phenomenon. The strong interaction between GO and AgNPs are negative charge of GO by the presence of carboxylate groups and positively charged AgNPs due to the presence of amine groups in the AgNPs.

Finally, we performed the Raman spectroscopy to characterize the structural and electronic properties of GO nanosheets and the interaction between GO and AgNPs, including the disorder, crystal structure, defects and doping levels of graphite, GO and graphene-based materials and nanocomposites [18,19,20] Figure 1F shows the Raman spectra of GO before and after AgNPs were absorbed on its surface. Two characteristic D and G bands of GO were observed at around 1381 cm^−1^ and 1587 cm^−1^, whereas GO-AgNPs exhibited the two characteristic D and G bands at around 1367 cm^−1^ and 1599 cm^−1^, respectively. The D band provides information on the breathing mode of the κ-point, and the G band relates to the tangential stretching mode of the E_2g_ phonon of the sp^2^ carbon atom [18,19,20,33]. Interestingly, the intensities of these two bands between GO and GO-AgNPs were enhanced significantly from 1.4 to 1.9 after the decoration of AgNPs on the surface of GO due to the surface-enhanced Raman spectroscopy (SERS) of AgNPs (Figure 1F). All these characterization results indicated the successful GO synthesis and decoration of AgNPs on the surface of graphene sheets using luciferin as reducing agents.

### 2.2. Dose Dependency of GO-AgNPs on Cell Viability and Proliferation of SH-SY5Y Cells

**Figure 2:**
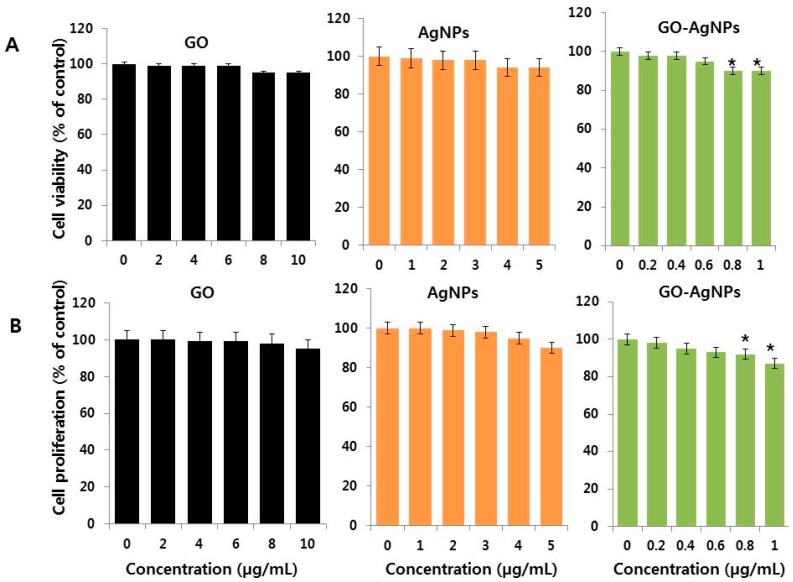
Effect of low concentration of GO-AgNPs on cell viability and proliferation of SH-SY5Y cells. (**A**) The viability of SH-SY5Y cells was determined after 24-h exposure to different concentrations of GO (2–10 µg/mL), AgNPs (1–5 µg/mL) and GO-AgNPs (0.2–1.0 µg/mL) using the CCK-8 assay; (**B**) Cell proliferation of SH-SY5Y cells was determined after 24-h exposure to different concentrations of GO (2–10 µg/mL), AgNPs (1–5 µg/mL) and GO-AgNPs (0.1–1.0 µg/mL) using trypan blue exclusion and BrdU assay. At least three independent experiments were performed for each sample. The results are expressed as the mean ± standard deviation of three independent experiments. The treated groups showed statistically significant differences from the control group by the Student’s *t*-test (* *p* < 0.05).

Many reports suggest that GO and GO derived family materials are benevolent due to their low toxicity as a function of their dimensions, concentration, and aggregation; the solvent used for preparation of GO; and type of reducing agents used for reduction [16,17,18]. Therefore, we were interested in studying the effect of non-toxic concentration of GO-AgNPs on cell viability of SH-SY5Y cells. The cells were treated with various concentrations of GO (2–10 µg/mL), AgNPs (1–5 µg/mL) and GO-AgNPs (0.2–1.0 µg/mL). As we expected, all tested nanomaterials had no effect on cell viability (Figure 2A). Similarly, the tested concentrations of GO, AgNPs and GO-AgNPs also had no effect on cell proliferation by trypan blue assay. Our results are consistent with previous studies reporting that various types of macrophages treated with GO up to 25 µg/mL show no significant viability and cell proliferation reduction, regardless of the exposure time [35,36,37,38,39,40]. Recently, Pelin et al. reported that the biocompatibility of various types of GO on skin keratinocytes; the results displayed significant cellular damage induced by few-layer graphene (FLG) and GOs only at high concentrations (>0 µg/mL and >1 µg/mL for FLG and GOs, respectively) after the cells were exposed for 72 h, with variable potencies depending on graphene-based materials (GBMs) oxidation state. Cell proliferation was assessed by trypan blue and BrdU in cells treated with GO (2–10 µg/mL), AgNPs (1–5 µg/mL) and GO-AgNPs (0.2–1 µg/mL) [40]. The results clearly indicated that low concentration of all these tested nanomaterials have no significant effect on proliferation (Figure 2B). Our data are consistent with recent report suggest that GO has no effect on cell proliferation or survival of differentiated cells but it enhances the transition of hemangioblasts to hemogenic endothelial cells. Cell viability and cell proliferation assay revealed that there is no significant toxicity was observed at tested concentrations, except slight toxicity at higher concentration thus we selected GO (10 µg/mL), AgNPs (5 µg/mL) and GO-AgNPs (1.0 µg/mL) for further experiments.

### 2.3. The Effect of Time on Cell Viability of SH-SY5Y Cells

**Figure 3:**
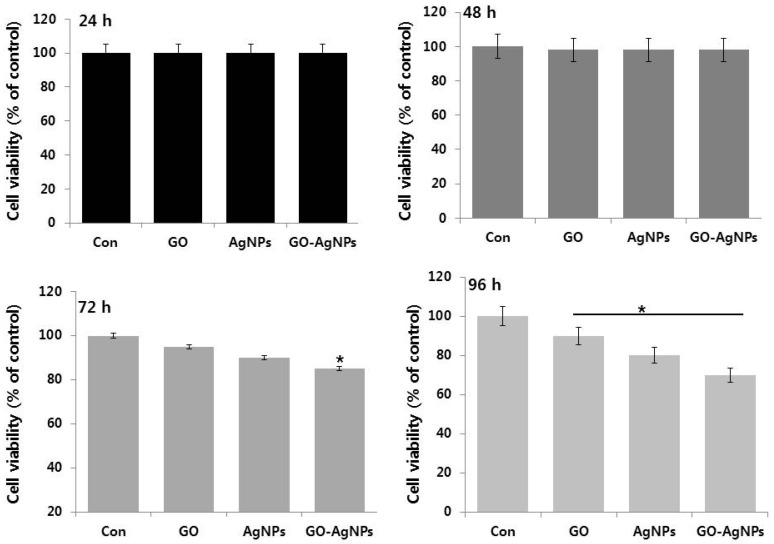
Time dependent effect on SH-SY5Y cells. The viability of SH-SY5Y cells was determined after 24-h exposure to GO (10 µg/mL), AgNPs (5 µg/mL) and GO-AgNPs (1.0 µg/mL) using the CCK-8 assay. At least three independent experiments were performed for each sample. The results are expressed as the mean ± standard deviation of three independent experiments The treated groups showed statistically significant differences from the control group by the Student’s *t*-test (* *p* < 0.05).

Next, we examined the effect of various time points on cell viability of SH-SY5Y cells. To quantify the effects of various time points, the cell growth rate was observed in the presence or absence of various nanomaterials such as GO (10 µg/mL), AgNPs (5 µg/mL) and GO-AgNPs (1.0 µg/mL) for 24, 48, 72 and 96 h (Figure 3). The results suggest that GO (10 µg/mL), AgNPs (5 µg/mL) and GO-AgNPs (1.0 µg/mL) did not exert any significant effect up to 48 h. However, when increasing time of incubation, trivial cell viability loss was observed. In agreement with previous reports, GO presented no cytotoxicity at a concentration lower than 80 µg/mL and it exhibited higher toxicity for SH-SY5Y cells when treated for the longer culture time at a concentration >80 µg/mL [17]. Hu et al. found that A549 cells remained viable with membrane integrity intact when cells were treated with culture medium containing 10% FBS and 100 µg/mL GO; that is GO can cause a dose-dependent oxidative stress in A549 cells but does not damage the cell membrane [41]. Our previous studies also confirmed that low concentration of GO has no effect on primary mouse embryonic fibroblast cells and human breast cancer cells [19]. Similarly, Dayem et al., reported that low concentration of AgNPs (0.2 µM) had no significant toxicity in SH-SY5Y cells [14]. A study was performed based on dose and time in both undifferentiated and differentiated human adipose-derived stem cells (hASC) cells using 10- and 20-nm AgNPs; the results revealed that no significant viability loss was observed in undifferentiated hASC. Similarly, the addition of either 10- or 20-nm AgNPs following hASC differentiation resulted in no significant decrease in viability of undifferentiated hASC at any concentration. Interestingly, the results from cytotoxicity assay of AgNPs revealed that both differentiated and undifferentiated human mesenchymal stem cells (hMSCs) treated with high silver concentrations (≥20 µg/mL) for 24 h showed cytotoxicity but not with low-concentration treatments (≤10 µg/mL). Altogether, the data from this study suggest that concentration of GO (10 µg/mL), AgNPs (5 µg/mL) or GO-AgNPs (1 µg/mL) had no significant toxicity in SH-SY5Y cells. Therefore, we selected 24 h treatment time for further experiments.

### 2.4. GO-AgNPs Enhances Differentiation of SH-SY5Y Cells

**Figure 4:**
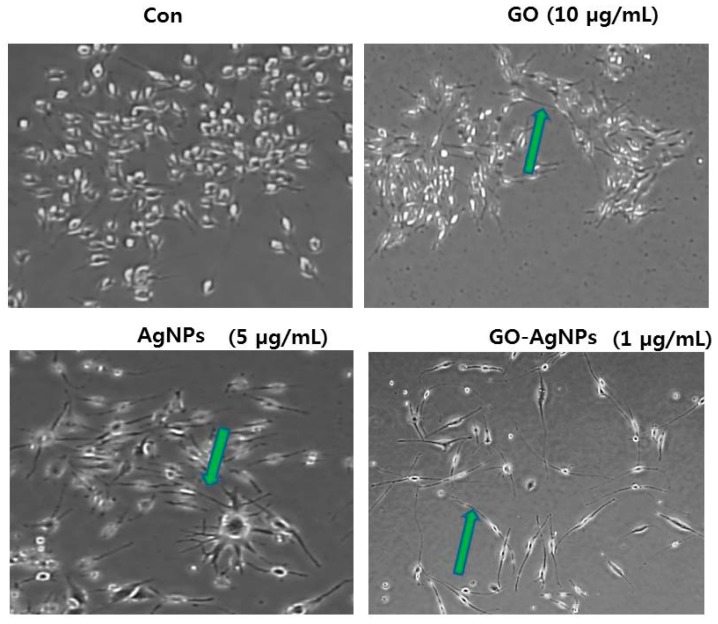
The effect of GO-AgNPs treatment on differentiation of SH-SY5Y cells. The GO-AgNPs induced differentiation of SH-SY5Y cells was determined after 24-h exposure to GO (10 µg/mL), AgNPs (5 µg/mL) and GO-AgNPs (1.0 µg/mL). Phase contrast microscopy images showing the morphological changes in SH-SY5Y cells after treatment with GO-AgNPs in 1% serum-supplemented medium. The green arrows indicate significant, lengthy neurite outgrowth. At least three independent experiments were performed for each sample. Scale bar is 100 μm.

To eliminate variables, such as time, in our experiments, first we studied the effect of low concentration GO (10 µg/mL), AgNPs (5 µg/mL) and GO-AgNPs (1 µg/mL) on differentiation of SH-SY5Y cells. We used low percentage of serum (1%) in the media [20]. The morphological assessment was analyzed by phase contrast microscope. As shown in Figure 4, the phase contrast images of SH-SY5Y cultures at control cells showed no particular neurite outgrowth and the cells have a round shape, whereas GO-treated cells showed significant number of short neurites (green arrows). Cells treated with AgNPs had significantly greater number of long neurites compared to GO, which were more numerous, more clustered and longer than the GO-induced neurites. The morphology of GO-AgNPs treated SH-SY5Y was relatively polar and cells grew faster and significantly longer than cells treated with other nanomaterials such as GO and AgNPs, which is distinguished and significantly better than other treatments. Interestingly, GO-AgNPs induced branching of longer neurites and detectable network formation. The data from our study corroborate with recently reported findings of SH-SY5Y cells using various differentiating agents such as all-*trans*-retinoic-acid (RA), estradiol (E2), cholesterol (CHOL), and brain-derived neurotrophic factor (BDNF) [27,28,29,30,31]. Teppola et al. observed that SH-SY5Y cells treated with RA, E2, CHOL, and BDNF induced various range of branching of shorter and longer neurites and detectable network formation [42]. Graphene acts as suitable substrate for differentiation process and induces the differentiation of human NSCs more toward neurons than glial cells [43]. It is known that GO could induce differentiation efficiency in the presence of RA in SH-SY5Y cells [17]. Graphene and its related materials induce stem cell differentiation into osteogenic, neuronal, and adipogenic lineages [44]. GO film provides a suitable environment for the adhesion, proliferation, and differentiation of human adipose derived stem cells (ADSCs) and GO film enhances the differentiation of human ADSCs to osteoblasts and adipocytes [45]. Graphene enhances viability, proliferation and differentiation of human mesenchymal stem cells (MSCs) and PC-12 cells [46,47]. Similarly, AgNPs induces neuronal differentiation via modulation of reactive oxygen species, phosphatases, and kinase signaling pathways in SH-SY5Y cells [14]. AgNPs serving as favorable anchoring sites and significantly enhanced neurite outgrowth [15]. Results from this study show, potentially for the first time, induced differentiation of SH-SY5Y cells under non-toxic concentration of GO-AgNPs. Surprisingly, with 24 h treatment at 1 µg/mL of GO-AgNPs, SH-SY5Y cells displayed morphological changes that became more evident for differentiation (Figure 4). The neuroblastoma cells acquired an apparent neuronal morphology with long extensions similar to those induced by all-trans retinoic acid (ATRA) treatment, the most commonly used protocol for differentiation of SH-SY5Y cells to more mature neuron-like phenotype.

### 2.5. GO-AgNPs Increase the Expression Level of Neuronal Markers in SH-SY5Y Cells

**Figure 5:**
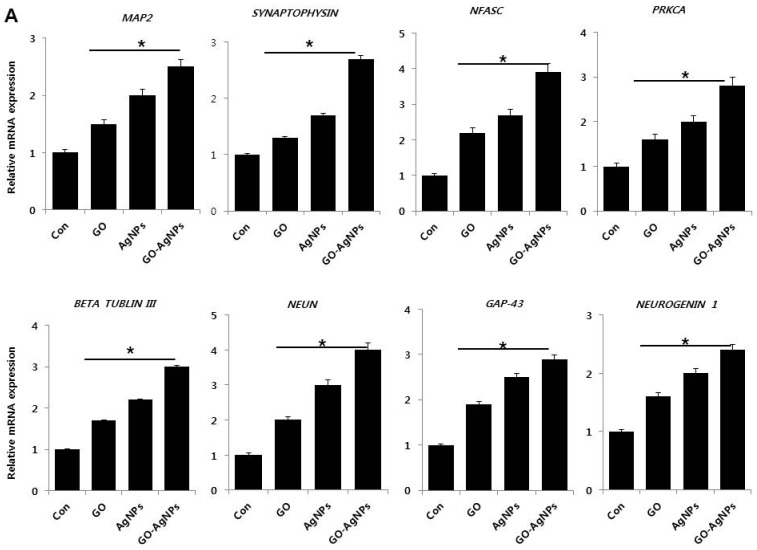

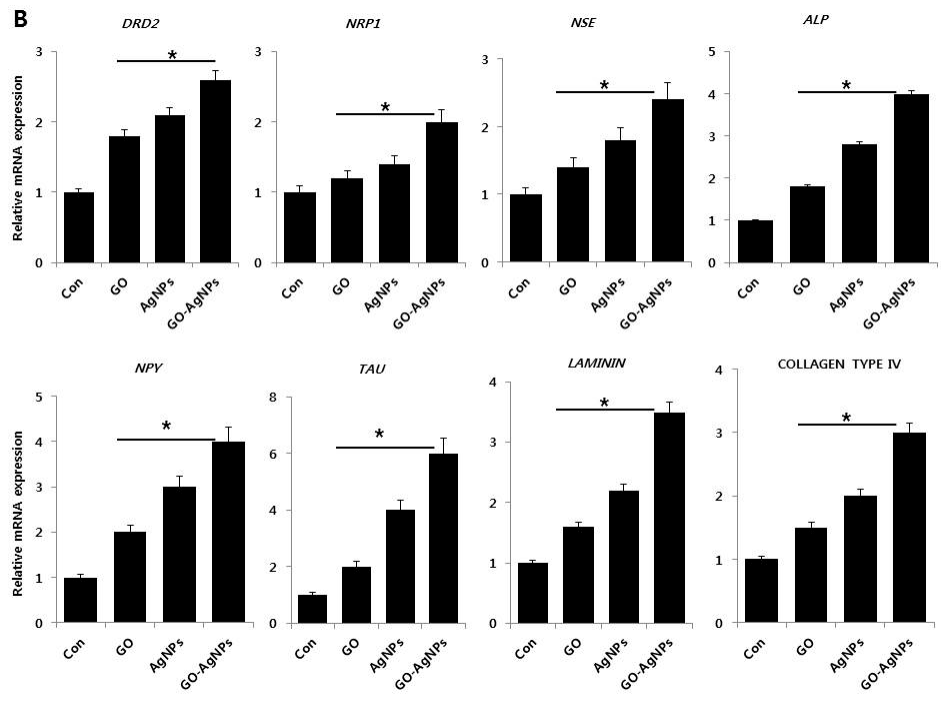
(**A**) Reverse transcription-quantitative polymerase chain reaction (RT-qPCR) analysis of expression of various neuronal markers. Microtubule-associated protein 2 (*MAP2*), synaptophysin, neurofascin (*NFASC*), protein kinase C, alpha (*PRKCA*), β tublin III, fox-1 homolog 3 (*NEUN*), growth associated protein 43 (*GAP-43*) and neurogenin 1, were analyzed after exposure of SH-SY5Y cells to GO (10 µg/mL), AgNPs (5 µg/mL) and GO-AgNPs (1.0 µg/mL) for 24 h. After 24 h treatment expression fold level was determined as fold changes in reference to expression values against GAPDH; (**B**) Reverse transcription-quantitative polymerase chain reaction (RT-qPCR) analysis of expression of various neuronal markers. The expression pattern of various neuronal markers genes such as dopamine receptors type 2 (*DRD2*), neuropilin 1 (*NRP1*), gamma neuronal (*NSE*), alkaline phosphatase (*ALP*), neuropeptide Y, (*NPY*), microtubule-associated protein tau (*TAU*), laminin, and collagen type IV were analyzed after exposure of SH-SY5Y cells to GO (10 µg/mL), AgNPs (5 µg/mL) and GO-AgNPs (1.0 µg/mL) for 24 h. After 24 h treatment expression fold level was determined as fold changes in reference to expression values against GAPDH. Results are expressed as fold changes. At least three independent experiments were performed for each sample. The results are expressed as the mean ± standard deviation of three independent experiments The treated groups showed statistically significant differences from the control group by the Student’s *t*-test (* *p* < 0.05).

To gain evidence on whether GO-AgNPs could stimulate the expression various differentiation responsible genes within the cells, qRT-PCR was performed to determine the expression of various neuronal markers in SH-SY5Y cells in the presence and absence of GO, AgNPs and GO-AgNPs. The expression of the following genes were studied: microtubule-associated protein 2 *(MAP2*), synaptophysin, neurofascin (*NFASC*), protein kinase C, alpha (*PRKCA*), *β tublin III*, fox-1 homolog 3 *(NEUN*), growth associated protein 43 (*GAP-43*), neurogenin 1, dopamine receptors type 2 (*DRD2*), neuropilin 1 (*NRP1*), gamma neuronal *(NSE*), alkaline phosphatase (*ALP*), neuropeptide Y, (*NPY*), microtubule-associated protein tau (*TAU*), laminin, and collagen type IV. The relative expression level of all tested genes was observed as from 0.2 to 6 fold after treatment with GO, AgNPs and GO-AgNPs. The expression level was dependent on type of nanomaterials (Figure 5A,B). However, GO-AgNPs upregulated all of the above genes in higher level compared to the cells treated with GO or AgNPs. It suggests that GO-AgNPs could promote neuronal differentiation of SH-SY5Y cells. Among several genes tested, typical neuronal markers such as *MAP2*, *TAU*, *NSE*, and *NEUN* were up-regulated in a stronger manner. Similarly, Graser et al. found expression of *MAP2*, *TAU*, *NSE*, and *NEUN* at a higher level in tissue-nonspecific alkaline phosphatase in the human SH-SY5Y neuroblastoma cell line [35]. Dayem et al. found that AgNPs enhanced the expression of neuronal differentiation markers including markers *Map-2*, *-β tubulin III*, *synaptophysin*, *neurogenin-1*, *Gap-43*, *and Drd-2*, in SH-SY5Y cells [14]. Yang et al. demonstrated that hierarchical topographies of specific dimensions of graphene oxide (GO)-based patterned substrate (GPS) influence the expression of neuronal markers such as *Tuj1* and *MAP2* gene expression in human neural stem cells (hNSCs). They found that the expression level was significantly higher on the GPS with a 5 µm groove width compared to cells cultured on flat SiO_2_ substrates (FS) or GO-coated substrates (GS) [48]. Garcia-Alegria et al. observed an increased level of the expression of the hematopoietic genes *Pu.1*, *MPO*, *CD45*, and *Gata1*, which suggest that GO could promotes embryonic stem cell differentiation to hematopoietic lineage [26]. Altogether, the present findings suggest that the combination of GO and AgNPs could synergistically induce neuronal differentiation by increasing expression of various neuronal markers genes, which supports the morphological observation of SH SY5Y cells.

### 2.6. GO-AgNPs Reduced the Expression Level of Stem Cell Markers in SH-SY5Y Cells

**Figure 6:**
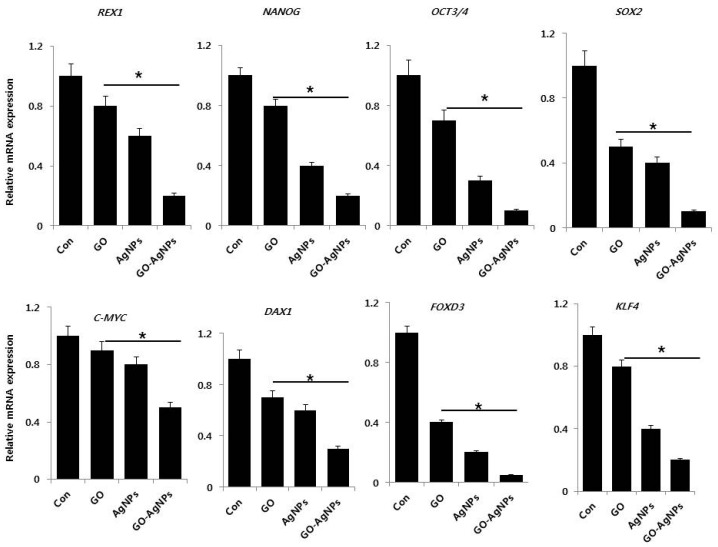
Reverse transcription-quantitative polymerase chain reaction (RT-qPCR) analysis of expression of various stem cell markers. The expression pattern of various stem cell markers genes were analyzed by exposure of SH-SY5Y cells to GO (10 µg/mL), AgNPs (5 µg/mL) and GO-AgNPs (1.0 µg/mL) for 24 h. After 24 h treatment, expression fold level was determined as fold changes in reference to expression values against GAPDH. Results are expressed as fold changes. At least three independent experiments were performed for each sample. The results are expressed as the mean ± standard deviation of three independent experiments The treated groups showed statistically significant differences from the control group by the Student’s *t*-test (* *p* < 0.05).

The expression of stem cell-related markers has been found in different cancers, including neuroblastoma [36]. However, the precise role of these stem cell-related genes in tumors is not clear, but Oct4 has been associated with a more immature and aggressive cell phenotype [37]. To further verify the effect of GO-AgNPs on neuronal stem cell markers, SH-SY5Y cells were treated with GO-AgNPs for 24 h and the gene expression of several well-known stem cell markers, including *REX1*, *NANOG*, *OCT3/4*, *SOX2*, *C-MYC*, *DAX1*, *FOXD3*, and *KLF4*, was assessed by qPCR. The expression of these stem cell markers was found to be downregulated (Figure 6). Among the tested various stem cell markers, except c-Myc all markers strongly down regulated. Collectively, these results suggest that GO-AgNPs is highly effective in decreasing the self-renewal capacity of SH-SY5Y cells, and in eliminating most neuroblastoma (NB) stem cells. Our findings are line with the observations of Mazzoccoli et al., who found a significant decrease of Oct4 in *N-*acetylaspartate (NAA)-treated SH-SY5Y cells [36]. Rex1 (zfp42) is a zinc finger protein expressed primarily in undifferentiated stem cells. When murine embryonic stem (ES) cells were treated with all-trans retinoic acid, the expression level of *Rex1* was decreased several fold [49]. *Nanog* is a potential gene for maintaining mouse ES cell self-renewal, which is also regulated by *FoxD3*, *p53* and *Oct4*. Furthermore, Nanog works together with other key pluripotent factors such as Oct4 and Sox2 to control a set of target genes that have important functions in ES cell pluripotency [50]. Hämmerle et al. reported that RA/MG132 reduces the expression level of the stemness markers such as Oct4, Sox2 and Nanog as well as the neural progenitor markers CD34 and Nestin in neuroblastoma cell lines [51]. Human N-Myc proto oncogene (*MYCN*) and avian myelocytomatosis virus oncogene cellular homolog (c-Myc) are critical player in the process of proliferation, differentiation and survival of hematopoietic stem cells (HSCs) and cerebellum progenitor cells. Furthermore, the deletion of endogenous MYCN and c-Myc in iPSCs potentially reduces self-renewal capacity, pluripotency and survival, followed by induction of differentiation [51,52,53,54,55]. The knock down of Dax-1, which is an orphan member of the nuclear hormone receptor superfamily induced upregulation of multi-lineage differentiation markers, and produced enhanced differentiation and defects in ES viability and proliferation [56]. FOXD3 plays important roles in biological processes, such as differentiation, proliferation, apoptosis and tumorigenesis [57]. Our results indicate that the significant down regulation of FOXD3 could play an important role in GO-AgNPs induced differentiation in SH-SY5Y cells. Shum et al. demonstrated that Krüppel-like factor 4 (KLF4) suppresses neuroblastoma cell growth; is involved in non-tumorigenic lineage differentiation; and is involved in activation of transcription of many target genes including *Nanog*, *Oct4*, *p21*, *p27*, *Rb*, and *Sox2*. GO-AgNPs significantly down regulate *KLF4* gene expression in SH-SY5Y cells [58] (Figure 6). Our data, consistent with previous studies, indicate the involvement of KLF4 in differentiation, and eventually reducing the proliferation of cancer cells. Our results revealed a significant down regulation of *Nanog*, *Oct3/4*, *Sox2* and other members of stem cell markers by GO-AgNPs, which could reduce the self-renewal capacity of SH-SY5Y cells and increase the differentiation. These observations suggest that GO-AgNPs represents a novel and specific inhibitor of pluripotency by changing gene expression pattern in SH-SY5Y shifted toward a more differentiated phenotype.

### 2.7. GO-AgNPs Induces Oxidative Stress and Suppresses Anti-Oxidative Stress Markers in SH-SY5Y Cells

**Figure 7:**
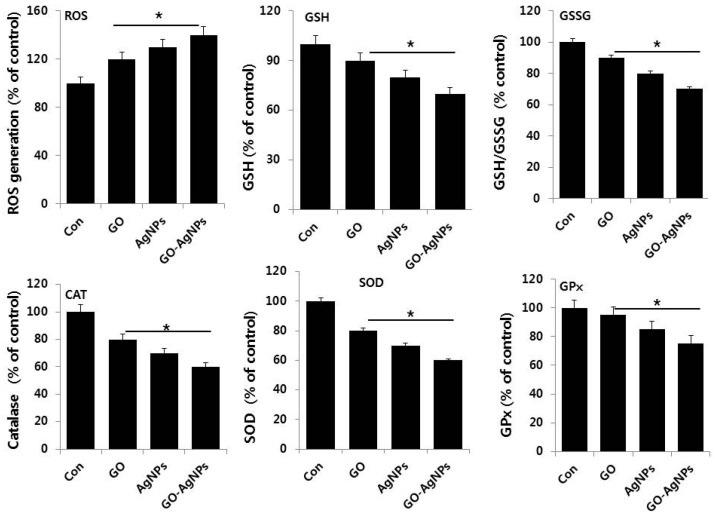
Effect of GO, AgNPs and GO-AgNPs on oxidative and anti-oxidative markers. The expression patterns of various oxidative and anti-oxidative markers were analyzed by exposure of SH-SY5Y cells to GO (10 µg/mL), AgNPs (5 µg/mL) and GO-AgNPs (1.0 µg/mL) for 24 h. After incubation, the cells were harvested, washed twice with ice-cold PBS, and then disrupted by ultrasonication for 5 min on ice. ROS generation was measured as described previously. The concentration of GSH and GSH/GSSG was expressed as milligram per gram of protein. The specific activity of catalase (CAT) was expressed as unit per milligram of protein. The specific activity of superoxide dismutase (SOD) was expressed as unit per milligram of protein. The specific activity of glutathione peroxidase (GPx) was expressed as unit per milligram of protein. The results are expressed as the mean ± standard deviation of three independent experiments The treated cells showed statistically significant differences from the untreated cells, as determined by the Student’s *t*-test (* *p* < 0.05).

Generally, reactive oxygen species (ROS) is a crucial player for oxidative stress induced cell death through modulation of various signaling pathways. Recent studies suggest that ROS may be involved in the differentiation of hematopoietic lineages or macrophage cell lines and low level of ROS is essential to activate signaling pathways to initiate biological processes and stem cell differentiation [59,60]. To understand the role of oxidative stress and anti-oxidative stress markers in differentiation of SH-SY5Y cells, the cells were treated with GO (10 µg/mL), AgNPs (5 µg/mL) and GO-AgNPs (1.0 µg/mL) for 24 h and the level of each marker was examined. As expected, GO, AgNPs and GO-AgNPs significantly induced ROS level compared to control; however, GO-AgNPs treated cells show significantly higher levels than GO and AgNPs (Figure 7). On the other hand, we were interested in measuring the level of anti-oxidative stress markers such as glutathione (GSH), glutathione disulfide (GSSG) superoxide dismutase (SOD), catalase (CAT), and glutathione peroxidase (GPx). Interestingly, all tested anti-oxidative markers levels were significantly lower than untreated cells. Consistent with our findings, previous study also demonstrated that all-*trans* retinoic acid (ATRA) increases ROS level, and subsequently the anti-oxidative stress marker GSH level was decreased. It has been reported that osteoblast differentiation is associated with a shift in redox potential to increased levels of oxidative stress, indicated by decreased total glutathione and GSH/GSSG ratio [61,62]. Accordingly, we found that GO-AgNPs treatment led to decreases in both total GSH content and ratio of GSH/GSSG, and the addition of GO-AgNPs caused further decreases in both indices. When GSH levels are transiently depleted, the availability of ROS is provisionally enhanced, leading to the activation of the cell differentiation program in human promyelocytic leukemia (HL-60) cells and this transient reduced level of GSH induced by GO-AgNPs could be reasonable factor for cell differentiation [63]. Therefore, GO-AgNPs treatment led to oxidative stress, i.e., a decrease in redox status, which favors neuronal cell differentiation. Further, to investigate the complete status of redox in SH-SY5Y cells in the presence of GO-AgNPs, we assessed anti-oxidants markers such as CAT, SOD, and GPx, which can counteract oxidative stress. Our findings suggest that all these tested markers were significantly decreased. Our findings corroborated with earlier finding suggest that decreased catalase activity is associated with increased differentiation of human neuroblastoma cell line (IMR-32) [64]. Upon the treatment with ATRA, the level of anti-oxidant markers including GSH, (GPx), and catalase levels were significantly decreased following ATRA treatment compared to untreated group in several time points in human SK-N-SH neuroblastoma cells [65,66]. Recently, Dayem et al. reported that moderate level ROS induced by AgNPs seems to be important factor for differentiation in SH-SY5Y cells [14]. Quinolinic acid induces neurite genesis in SH-SY5Y coincided with an increase in the generation of ROS [67]. Altogether, our findings suggest that redox biology in GO-AgNPs treatments could stimulate expression of neuronal differentiation markers in neuroblastoma cells and indicate that GO-AgNPs use different mechanistic approaches to induce differentiation in SH-SY5Y cells. The results from our study clearly demonstrate that GO-AgNPs induced level of ROS and decreased level of anti-oxidants activities involved in neuronal differentiation in SH-SY5Y cells. The results from this study suggest that the possible mechanisms by which GO-AgNPs nanocomposites upregulate are due to an imbalance between the production of reactive oxygen species and anti-oxidant level and the ability of the biological system to readily detoxify the reactive intermediates or easily repair the resulting damage. The excess formation or insufficient removal of highly reactive molecules, such as reactive oxygen species (ROS), and resultant oxidative stress can arise from an increase in oxidant generation, a decrease in antioxidant protection, or a failure to repair oxidative damage. These are possible mechanisms of differentiation induced by GO-AgNPs.

### 2.8. GO-AgNPs Induce the Expression of Various Signaling Molecules for Differentiation

**Figure 8:**
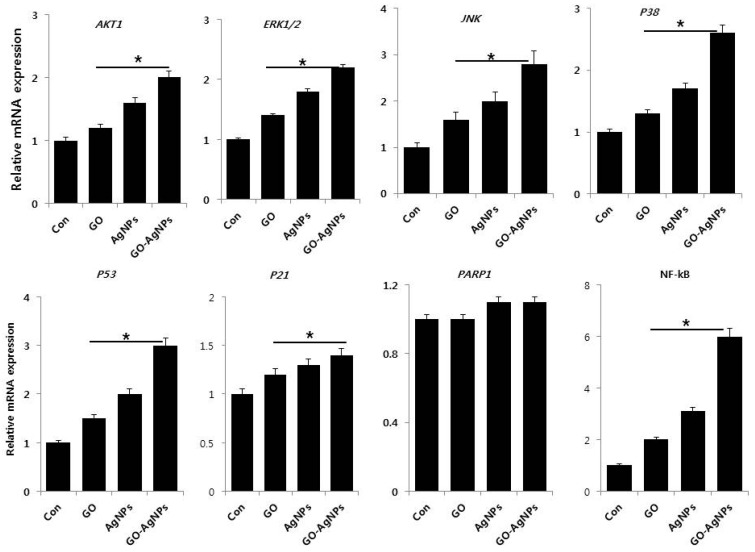
Reverse transcription-quantitative polymerase chain reaction (RT-qPCR) analysis of expression of various genes involved in signaling pathways. The expression pattern of various genes involved in signaling pathways were analyzed by exposure of SH-SY5Y cells to GO (10 µg/mL), AgNPs (5 µg/mL) and GO-AgNPs (1.0 µg/mL) for 24 h. After 24 h treatment, expression fold level was determined as fold changes in reference to expression values against GAPDH. Results are expressed as fold changes. At least three independent experiments were performed for each sample. The results are expressed as the mean ± standard deviation of three independent experiments The treated groups showed statistically significant differences from the control group by the Student’s *t*-test (* *p* < 0.05).

Cell differentiation is a complex process regulated by signaling molecules, including hormones, growth factors, cytokines, trophic factors, and morphogens. For instance, RA treatment activates the PI3K/Akt signaling pathway [68]. To investigate the role of various genes involved in critical signaling pathway in differentiation, *AKT1*, *ERK1/2*, *JNK*, *P38*, *P53*, *P21*, *PARP1* and *NF-κB* were analyzed by qPCR in SH-SY5Y cells exposed to GO (10 µg/mL), AgNPs (5 µg/mL) and GO-AgNPs (1.0 µg/mL) for 24 h. The results from this study clearly indicated that all tested genes were upregulated except *PARP1* by GO-AgNPs. The expression levels of all tested genes were upregulated 1–6 fold, depending on type of gene and type of nanomaterial. Among the tested nanomaterials, GO-AgNPs strongly promoted the expression of all tested genes, which is due to the two different unique physical and chemical properties of nanomaterials in the single platform and working synergistically. Our findings gained support from a previous study suggesting that AgNPs could induce neuronal differentiation via modulation of ROS, phosphatases, and kinase signaling pathways such as Akt and Erk1/2 in SH-SY5Y cells and oligosaccharide esters of tenuifoliside A promotes neurite outgrowth in PC12 cells via activation of various signaling pathways including mitogen-activated protein kinase kinase (MEK),extracellular signal-regulated kinase (ERK), cyclic adenosine monophosphate (cAMP) response element binding (CREB) and Phosphatidylinositol-3-Kinase and Protein Kinase B (PI3K/Akt) [14,69]. Tetramethylpyrazine (TMP) induces differentiation in SH-SY5Y cells by expression of ERK1/2 and PI3K/Akt by modulating TopoIIβ expression [70]. The exposure of both undifferentiated and differentiated SH-SY5Y cells to 1-methyl-4-phenylpyridinium (MPP+) exhibited increased expression of Akt1, Akt2, and Akt3 [71]. The activation of neuronal differentiation by pituitary adenylate cyclase-activating polypeptide (PACAP-38) and forskolin stimulated the activation of extracellular signal-regulated kinase (ERK), mitogen-activated protein kinase (MAP; p38 MAP kinase) and c-Jun N-terminal kinase (JNK) was confirmed by the MEK1 inhibitor PD98059 and the p38 MAP kinase inhibitor SB203580. Yu et al. observed that c-Jun N-terminal kinase 1 (JNK1) plays an essential role in RA-induced neurite outgrowth of SH-SY5Y cells [72]. Graphene oxide promotes differentiation by upregulation of focal adhesion kinase (FAK) and Erk1/2 in human adipose derived stem cells [73]. *P53*, *P21*, *PARP1* and *NF-κB* are involved not only in apoptosis but also involved in wide range of other biological processes, such as differentiation, metabolism, fecundity, and aging [74]. ATRA can induce apoptosis by the activation of the extrinsic and p53 pathways in SH-SY5Y and SK-N-Be (2) cells [75]. It is well known that p53 upregulates p21 in various cell lines when the cells are treated with cytotoxic agents. Our findings, consistent with previous observations, demonstrated the expression of a higher basal level of p21WAF1 in differentiated cells of NBLW and NB69 compared to undifferentiated control cells (Figure 8) [76]. NF-κB/Rel proteins play an important role in synaptic transmission and neuronal plasticity as well as neuronal development and differentiation [77,78]. Consistent with previous studies on differentiation induced by other molecules, GO-AgNPs could induce the expression of NF-κB in SH SY5Y cells during differentiation. Altogether, the evidence gained from this study clearly indicates the mechanisms of differentiation by GO-AgNPs are regulated through modulation of various signaling molecules involved in intracellular pathways.

The mechanism of cell death caused by AgNPs and graphene oxide are well known in various cancer and non-cancer cells by inducing oxidative stress, whereas the mechanism of differentiation caused by graphene oxide–silver nanoparticles remains obscure. This is the first study demonstrating that the important functional role of GO-AgNPs in differentiation, which is depends on the level of oxidative stress, dose and time. Interestingly, this study revealed that a low concentration of GO-AgNPs induces cell differentiation by upregulation of neuronal differentiation markers and down regulation of stem cell markers as well as the modulation of various signaling molecules involved in cell survival, cell proliferation, cell death and differentiation by induction of low level of oxidative stress. It is well known that various biological processes are activated by low levels of ROS via activation of different signaling pathways involved in normal physiological and biological processes and also low level of ROS induces differentiation by maintaining the balance between pro-oxidant and anti-oxidant levels. Particularly, the size, shape, surface area and surface chemistry of nanoparticles play important roles in cell toxicity/differentiation. Although our data clearly demonstrate that differentiation processes are due to the level of oxidative stress influenced by silver ion and oxidation of efficiency of graphene oxide, characterization of physicochemical properties of internalized GO-AgNPs remains a future challenge, which, is necessary to further unravel the pathways of differentiation and their attribution to distinct physicochemical properties of GO-AgNPs.

## 3. Materials and Methods

### 3.1. Materials

Penicillin–streptomycin solution, trypsin-ethylenediaminetetraacetic acid (EDTA) solution, Dulbecco’s Modified Eagle’s Medium (DMEM), and 1% antibiotic-antimycotic solution were obtained from Life Technologies (GIBCO, Grand Island, NY, USA). Fetal bovine serum (FBS), CCK-8, and an in vitro cell-counting assay kit were purchased from Dojindo (ck04; Rockville, MD, USA). Luciferin, AgNO_3_, and the in vitro toxicology assay kit were purchased from Sigma-Aldrich (St. Louis, MO, USA). Graphite (Gt) powder, NaOH, KMnO_4_, NaNO_3_, anhydrous ethanol, 98% H_2_SO_4_, 36% HCl, 30% H_2_O_2_ aqueous solution, and all other chemicals were purchased from Sigma-Aldrich unless otherwise stated.

### 3.2. Synthesis of GO

GO was synthesized as described previously with suitable modifications [79,80,81]. In a typical synthesis process, 2 g natural Gt powder was added to 350 mL H_2_SO_4_ at 0 °C; 8 g KMnO_4_ and 1 g NaNO_3_ were added gradually while stirring. The mixture was transferred to a 40 °C water bath and stirred for 60 min. Deionized water (250 mL) was slowly added, and the temperature was increased to 98 °C. The mixture was maintained at 98 °C for 30 min; the reaction was terminated by the addition of 500 mL deionized water and 40 mL 30% H_2_O_2_ solution. The color of the mixture changed to brilliant yellow, indicating the oxidation of pristine Gt to Gt oxide. The mixture was filtered and washed with diluted HCl to remove metal ions. The product was washed repeatedly with distilled water until a pH of 7.0 was achieved; the synthesized Gt oxide was further sonicated for 15 min. To prepare smaller size of GO, the as prepared brownish mixture of GO was centrifuged and washed several times with deionized water. To obtain uniform distribution of smaller size of GO sheets, a low-speed centrifugation was performed at 5000 rpm for 15 min to remove thick multilayer flakes until all the visible particles were removed. Then the supernatant was further subjected to ultrasonication for 6 h. The resulting GO samples were used for further analysis.

### 3.3. Synthesis of AgNPs

Synthesis of AgNPs was carried out according to a method described previously [82]. AgNPs were synthesized by incubating 1 mg/mL of luciferin (Sigma-Aldrich) in 100 mL of water containing 5 mM AgNO_3_ at 37 °C for 12 h. The color change from colorless to reddish brown in color can be attributed to the formation of AgNPs in the reaction mixture. Further characterizations of the synthesized AgNPs were performed as described previously [44]. 

### 3.4. Synthesis and Characterization of GO-AgNPs Nanocomposite

Synthesis and characterization of the GO-AgNPs nanocomposite was followed as described previously [83]. GO-AgNPs nanocomposites were prepared using luciferin. Aqueous solutions of 100 mg GO and 5 mM AgNO_3_ were used as precursors for the GO-AgNPs nanocomposites. Initially, 100 mg GO was dispersed in 60 mL water by 60 min of sonication. The reaction mixture was prepared in a 250-mL round-bottom flask by dissolving 5 mM AgNO_3_ in 30 mL water. To this solution, 60 mL of the GO dispersion was added, followed by a quick addition of 10 mL of aqueous luciferin at the final concentration of 1 mg/mL. The mixture was stirred at 90 °C for 12 h. After 12 h, the reaction was stopped, and the resultant mixture was washed three times with water using centrifugation. The product was obtained as a pure and used for further experiments. All the samples were characterized as prescribed previously [82,83]

### 3.5. Cell Culture and Exposure of SH-SY5Y Cells to GO-AgNPs Nanocomposite

SH-SY5Y human neuroblastoma cells were obtained from the American Type Culture Collection and were maintained in DMEM supplemented with 2 mM glutamine, 10% FBS, and 1% antibiotic-antimycotic solution. Cells were grown to confluence at 37 °C in a 5% CO_2_ atmosphere. All experiments were performed in 96-well plates and 100-mm cell culture dishes based on requirements. The cells were prepared in 100-µL aliquots at a density of 1 × 10^5^ mL and plated in 96-well plates. After the cells were cultured for 24 h, the medium was replaced with a medium containing various concentrations or required concentrations of GO or AgNPs or GO-AgNPs. After incubation for an additional 24 h, the cells were analyzed for viability. The cells that were not exposed to GO, AgNPs or GO-AgNPs served as controls. For all differentiation experiments, SH-SY5Y cells were grown in the same culture media supplemented with 1% FBS for 24 h and then washed with fresh media and further incubated with GO (10 µg/mL), AgNPs (5 µg/mL) or GO-AgNPs (1 µg/mL) for another 24 h.

### 3.6. Cell Viability Assay

The cell viability was measured with a CCK-8 cell-counting assay kit. Briefly, the SH-SY5Y cells were plated onto 96-well flat-bottom culture plates with various concentrations of GO or AgNPs or GO-AgNPs. All cultures were incubated for 24 h at 37 °C in a humidified incubator. After 24 h of incubation at 37 °C and 5% CO_2_ in a humid atmosphere, 10 µL of CCK-8 reagent was added to each well, and then the plates were incubated for a further 1 h at 37 °C. The resulting formazan absorbance was measured at 450 nm with an ELISA reader (Spectra MAX; Molecular Devices, Sunnyvale, CA, USA). The results were expressed as the mean of three independent experiments.

### 3.7. Cell Proliferation Assay

Cell proliferation was evaluated using the trypan blue assay as described earlier [84,85]. SH-SY5Y cells were plated in 6-well plates (2 × 10^5^ cells per well) and incubated for 24 h with various concentrations of GO or AgNPs or GO-AgNPs. Cells were cultured in the medium without GO or AgNPs or GO-AgNPs were used as controls. Twenty-four hours later, cells were detached with 300 µL trypsin–EDTA solution and both adherent and cells in suspension were collected. The mixture of the supernatant and detached cells was centrifuged at 1200 rpm for 5 min. The pellet was added to 700 µL of trypan blue solution and dispersed. After 5 min of staining, cells were counted using a cytometer. The viable cells were unstained, whereas dead cells were stained in blue. Three independent experiments were performed in triplicate. The mean and standard deviation were calculated. Cell proliferation was expressed as the percentage of viable cells.

### 3.8. BrdU Cell Proliferation Assay

Cell proliferation was determined according to method described earlier [85] and also manufacturer’s instructions (Thermo Fisher, Waltham, MA, USA). Cells were incubated with various concentrations of GO, AgNPs or GO-AgNPs for 24 h; the BrdU labeling solution was added to the culture medium 2 h before the end of the incubation. Cells were fixed and the level of incorporated BrdU was determined using the Cell Proliferation ELISA BrdU assay kit (Roche, Basel, Switzerland) following the manufacturer’s instructions. Proliferation activity of the untreated cells at the time point of 0 h was considered as 100%.

### 3.9. Determination of Reactive Oxygen Species (ROS)

ROS were estimated according to a method described previously [85]. Intracellular ROS were measured based on the intracellular peroxide-dependent oxidation of 2′,7′-dichlorodihydrofluorescein diacetate (DCFH-DA, Molecular Probes, Eugene, OR, USA) to form the fluorescent compound 2′,7′-dichlorofluorescein (DCF), as previously described. The cells were seeded onto 24-well plates at a density of 5 × 10^4^ cells per well and cultured for 24 h. After washing twice with PBS, fresh media containing GO or AgNPs or GO-AgNPs for 24 h was added and incubated for 24 h. The cells were then supplemented with 20 µM DCFH-DA, and the incubation continued for 30 min at 37 °C. The cells were rinsed with PBS, and 2 mL of PBS was added to each well. The fluorescence intensity was determined using a spectrofluorometer (Gemini EM, Molecular Devices, Sunnyvale, CA, USA) with excitation at 485 nm and emission at 530 nm.

### 3.10. Measurement of Oxidative and Anti-Oxidative Stress Markers

Oxidative stress markers, such as glutathione (GSH), glutathione disulfide (GSSG) superoxide dismutase (SOD), catalase (CAT), and glutathione peroxidase (GPx) activity were assayed with reagents from various kits, according to each manufacturer’s instructions (Sigma-Aldrich). Briefly, the cells were cultured in 75 cm^2^ culture flasks and exposed to GO or AgNPs or GO-AgNPs for 24 h. The cells were harvested in chilled PBS, by scraping and washing twice with 1× PBS at 4 °C for 6 min at 1500 rpm. The cell pellet was sonicated at 15 W for 10 s (3 cycles) to obtain the cell lysate. The resulting supernatant was stored at −70 °C, until analyzed.

### 3.11. Reverse Transcription-Quantitative Polymerase Chain Reaction (RT-qPCR) Assay

Total RNA was extracted from the cells treated with GO or AgNPs or GO-AgNPs for 24 h using the Arcturus PicoPure RNA isolation kit (Arcturus Bioscience, Mountain View, CA, USA), and then samples were prepared according to the manufacturer’s instructions. Real-time RT-PCR was conducted using a Vill7 (Applied Biosystems, Foster City, CA, USA) and SYBR Green as the double-stranded DNA-specific fluorescent dye (Applied Biosystems). Target gene expression levels were normalized to *GAPDH* expression, which was unaffected by treatment. The RT-PCR primer sets are shown in Tables S1–S3. Real-time quantitative RT-PCR was performed independently in triplicate for each of the different samples; the data are presented as the mean values of gene expression measured in treated samples versus control [85].

### 3.12. Statistical Analyses

All assays were conducted in triplicate, and each experiment was repeated at least three times. The results are presented as means ± standard deviation. All experimental data were compared using the Student’s *t*-test. A *p*-value less than 0.05 was considered statistically significant.

## 4. Conclusions

Graphene and graphene related materials are being utilized in various biomedical applications such as drug delivery, imaging, and cancer therapy. To explore novel biomaterial, GO-AgNPs were synthesized as a single platform containing two different nanomaterial, i.e., graphene, and silver by simultaneous reduction of GO and Ag^+^ in the presence of luciferin. This study, for the first time, demonstrates the use of GO-AgNPs prepared using luciferin as a reducing agent to promote differentiation of neuroblastoma cancer cells. The prepared GO-AgNPs exhibited differential functions in concentration dependent manner. Using low concentration of GO-AgNPs induces differentiation. The differentiation induced by GO-AgNPs was confirmed by series of cellular and biochemical assays, and gene expression analysis. Interestingly, low level of ROS is favorable for the process of differentiation. Herein, we demonstrated the functional aspects of GO-AgNPs at the mRNA level of genes involved in differentiation and self-renewal, suggesting the involvement of gene alterations. This study provides possible mechanism of redox biology and modulations of genes are involved in differentiation, self-renewal and important signaling pathways such as cell survival, cell death and differentiation process. However, further studies are warranted to give insight into the molecular mechanism of how GO-AgNPs act on tumor cells differentially.

## Supplementary Materials

Supplementary materials can be found at www.mdpi.com/1422-0067/18/12/2549/s1.
