# Supplementary material for: Graphene Oxide–Silver Nanoparticles Nanocomposite Stimulates Differentiation in Human Neuroblastoma Cancer Cells (SH-SY5Y)
Source: Int J Mol Sci. 2017 Nov 28;18(12):2549. doi: 10.3390/ijms18122549 (PMC5751152; doi:10.3390/ijms18122549)
Supplement: Supplementary file 1 [file ijms-18-02549-s001.pdf]

Graphene Oxide–Silver Nanoparticles Nanocomposite Stimulates Differentiation in Human Neuroblastoma Cancer Cells (SH-SY5Y)

Sangiliyandi Gurunathan * and Jin-Hoi Kim *

Department of Stem Cell and Regenerative Biotechnology, Konkuk University, Seoul 05029, Korea

* Correspondence: gsangiliyandi@yahoo.com (S.G.); jhkim541@konkuk.ac.kr (J.-H.K.); Tel.: +82-2-450-0581 (S.G.); +82-2-450-3687 (J.-H.K.)

Table S1

Gene	Primer
MAP2	F:CATGGGTCACAGGGCACCTATTC
	R:GGTGGAGAAGGAGGCAGATTAGCTG
NFASC	F:TCCTGGGCAAAGCTGAAAAC
	R:AGACGGTGAGTTTCAGGGAG
PRKCA	F:TATCGCCCCAGAGATAATCG
	R:CCTTGGACAAGGATTTTGGA
NEUN	F:CATGGGTCACAGGGCACCTATTC
	R:GGTGGAGAAGGAGGCAGATTAGCTG
GAP-43	F:AGAGGAACCTGAGGCTGACC
	R:GCTAGTGGGTGGGAAAGGAC
NEUROGENIN-1	F:GACCCTGTTTTCTCCTTCCC
	R:CCATCTATTGCCTGCTGACTAG
DRD-2	F:GTCATGATCTCCATCGTCTGG
	R:AATGAAGGGCACGTAGAAGG
NRP1	F:GAAGCACCGAGAGAACAAGG
	R:GTTGCCCTCAAAAGACTTCG
NSE	F:CCCAGAACTTCCCTGATTGA

	R:AAGTGGAAGACACGTGGGAC
ALPL	F:GCTGAACAGGAACAACGTGA
	R:AGACTGCGCCTGGTA
NPY	F:GCTGCGACACTACATCAACC
	R:CACCACATTGCAGGGTCTTC
TAU	F:AAGGTGACCTCCAAGTGTGG
	R:TATTGTCCAGGGACCCAATC
LAMININ B1	F:ACAACACCAAAGGCCTGAAC
	R:TGCCAGTAGCCAGGAAGACT
COLLAGENASE TYPE IV	F:AAAGGGAGAAAGAGGCTTGC
	R:CCTTTGTACCGTTGCATCCT

Table S2

Gene	Primer
REX1	F:ACTGGTACCTCGGATTTCAAATGGAGAGGTCCTGC
	R:AATCTGGCTAGCAGTGGAAACGTGGACTGCCCTGCG
NANOG	F:TCTTCCTTCCTCCATGGATCT
	R:AGGACTGGATGTTCTGGGTCT
OCT3/4	F:AGTGAGCAACCTGGAGA
	R:TTTTTCGTCGCTTGGAGACT
c-MYC	F:GGGGCTTTATCTAACTCGCTGTAG
	R:AGTCGTAGTCGAGGTCATAGTTCC
DAX1	F:GAGTCTGAACATCAGTACCAAGGA ,
	R:GCTCTTTATTCTTCCCTCATGGTG
FOXO3	F:GTCGTTCAGCATCGAGAACA
	R:AAATTGGGGAGAGGCAGAGT
KLF4	F:AGATGTCTAAGGAGCTGGTTGAGT
	R:GTACGCTCTGTGGATTCCTTTAGT

Table S3

Gene	Primer

**AKT1**	**F:AGGTGACACTATAGAATAGAGGAGATGGACTTCCGGTC**
	**R:GTACGACTCACTATAGGGAAGGATCTTCATGGCGTAGTAGC**
**ERK1/2**	**F:AGGTGACACTATAGAATAGGAGCAGTATTACGACCCGA**
	**R:GTACGACTCACTATAGGGAGATGTCTGAGCACGTCCAGT**
**JNK**	**F:AGGTGACACTATAGAATACAGAAGCTCCACCACCAAAGAT**
	**R:GTACGACTCACTATAGGGAGCCATTGATCACTGCTGCAC**
**p38**	**F:AGGTGACACTATAGAATATTCAGTCTTTGACTCAGATGCC**
	**R:GTACGACTCACTATAGGGAGTCAGGCTTTTCCACTCATCT**
**p53**	**F:AGGTGACACTATAGAATAGGGGAGCAGGGCTCA ,**
	**R:GTACGACTCACTATAGGGAAAAATGGCAGGGGAGGG**
**p21**	**F:AGGTGACACTATAGAATATTAGCAGCGGAACAAGGAGT**
	**R:GTACGACTCACTATAGGGAAGCCGAGAGAAAACAGTCCA**
**PARP1**	**F:AGGTGACACTATAGAATATATCGAGTCGAGTACGCCAA**
	**R:GTACGACTCACTATAGGGAGTGTGGGACTTTTCCATCAAA**
**NF-kB**	**F:AGGTGACACTATAGAATAGCGGGCGTCTAAAATTCTG**
	**R:GTACGACTCACTATAGGGATTCCACGATCACCAGGTAGG**
